# Health professional views on the assessment and management of foot problems in people with psoriatic arthritis in Australia and New Zealand: a qualitative investigation

**DOI:** 10.1186/s12891-019-2572-6

**Published:** 2019-05-04

**Authors:** Kate Carter, Steven Walmsley, Keith Rome, Deborah E. Turner

**Affiliations:** 1 Podiatry department, School of Science and Health, Western Sydney University, Building 24, Campbelltown Campus, Sydney, Australia; 2 Health and Rehabilitation Research Institute, Faculty of Health and Environmental Science, AUT University, 90 Akoranga Drive, Northcote, Auckland, 0627 New Zealand

**Keywords:** Psoriatic arthritis, Foot problems, Health professionals, Qualitative

## Abstract

**Background:**

Active foot disease persists in a high proportion of people with psoriatic arthritis despite the availability of pharmacological and non-pharmacological interventions to modify the course of the disease. Limited information exists on the provision of health care for foot disease in psoriatic arthritis. The objective of this study was to explore the views of health professionals on the assessment and management of people with psoriatic arthritis-related foot involvement.

**Methods:**

Convenience sampling was used to recruit health professionals working in rheumatology outpatient clinics in Sydney, Australia and Auckland, New Zealand. Three focus groups were undertaken to explore the views and experiences of health professionals on the assessment and management of foot problems in people with psoriatic arthritis. All interviews were audio-recorded and transcribed verbatim. Qualitative data was analysed using a constant comparative analytic approach to identify themes.

**Results:**

A total of seventeen health professionals participated including rheumatologists, podiatrists and a physiotherapist. Key themes derived from the focus groups suggest that health professionals perceived that people with psoriatic arthritis-related foot problems experience suboptimal management from symptom onset, to diagnosis and treatment. Frustration was expressed throughout discussions relating to lack of appropriate training and expertise required for the specialised management of foot problems typically encountered with psoriatic arthritis and poor access for patients to specialist podiatry services.

**Conclusions:**

This study provides new insight into the perspectives of health professionals on the management of foot problems related to psoriatic arthritis. Deficiencies in the diagnosis, assessment and treatment of foot problems were revealed. To meet the foot health needs of people with psoriatic arthritis, reducing diagnostic delay, improving knowledge and awareness about the disease among people with psoriatic arthritis and health professionals, and increasing specialist podiatry service provision may be required.

## Background

Psoriatic arthritis (PsA) is a chronic inflammatory disease characterised by a variety of musculoskeletal and dermatological manifestations [1]. The heterogeneity of clinical features makes the diagnosis and management of PsA difficult [1–3]. Major challenges recognised in previous studies on the management of PsA, which include under-diagnosis, diagnostic delays and under-treatment [4–7], are reflected in reports of high foot disease burden associated with PsA [8]. Despite intensive pharmacological management, imaging studies have shown that inflammation in the foot is detectable in a high proportion of people with PsA [9–11]. Clinically important levels of foot-related impairment and disability have been identified in those with localised inflammatory features in the foot affected by PsA [8].

Despite UK podiatry services being well established in the public health system, one UK-based study found that the majority of people with PsA reported foot pain and had not received professional foot care [8]. UK podiatrists have developed extended scope practices in rheumatology that include specialist training in corticosteroid injection therapy, musculoskeletal diagnostic ultrasound, gait analysis and rehabilitation [12]. However, it is generally perceived that Australia and New Zealand podiatrists have limited role extension and limited service provision in the public health system and therefore the severity of PsA-related foot disease reported in the UK may not represent those in other countries.

Previous studies conducted in Australia [13, 14] and New Zealand [15] suggest there is inadequate provision of podiatry services and significant unmet demand for foot care amongst people with rheumatoid arthritis (RA). It is possible that barriers to foot care exist for people with PsA, but the challenges specific to this patient group have not been investigated. Currently there is limited evidence to support the management of PsA specific foot problems [16]. Expert-led recommendations for PsA advocate the integration of podiatry within rheumatology multidisciplinary teams for rapid access to specialist foot care [1, 16]. However, little is known about the assessment and treatment of foot problems in PsA in Australia and New Zealand. The objective of this study was to explore the views of health professionals on the assessment and management of people with PsA-related foot involvement.

## Methods

### Study design

A qualitative research approach was chosen to identify concepts important to health professionals and to be able to explore and understand their views. Focus groups were used to provide a rich and deep examination of the experiences of health professionals through semi-structured, facilitated discussion. Sample size for each focus group was based on recommendations suggesting that 4–12 people will generate sufficient data [17]. Questions relating to the assessment and management of foot problems specific to PsA formed the interview guide (Table 1). These questions were developed based on a review of relevant literature [14, 18, 19] and were identified as being important by the research group, which comprised clinicians and academics.

**Table 1:** Focus group interview guide for health professionals with experience of assessing and managing people with psoriatic arthritis-related foot problems

	Exemplar questions	Prompts
1	How often do you examine the feet of patients with psoriatic arthritis?	Why do you think that is?
2	Have you encountered any barriers in relation to your patients receiving appropriate foot care?	Do patients seek help with foot problems? From whom? Have your patients reported any barriers to accessing appropriate foot care services to you? What factors do you think make it difficult for patients to access appropriate foot care?
3	Is there anything else you would like to add concerning the experiences of your patients in relation to their foot health and care?	

### Participants

A convenience sampling strategy was used to recruit health professionals from rheumatology outpatient clinics in Sydney, Australia and Auckland, New Zealand. Participating sites were selected to include health professionals from public and private sectors in hospital and community-based services, from lower and higher socioeconomic geographical areas and to provide local data from Australia and New Zealand. Health professionals with clinical experience of managing people with PsA, working in Australia or New Zealand were eligible for inclusion [20]. Potential participants were recruited by response to an invitation email containing an outline of the study, screening form and contact details of the primary researcher (KC).

### Procedure

Prior to the focus groups, demographic data was recorded including gender, ethnicity, occupation and the number of years of clinical experience. The focus groups were conducted by the same researcher (KC) and supported by a second investigator (SW). All focus groups were audio-recorded and transcribed verbatim immediately after each session. Data was collected between October 2017 and March 2018.

### Data analysis

Demographic data was summarised using descriptive statistics. Focus group transcripts were anonymised and imported into a data analysis software package (ATLAS-ti version 7.5.7 Scientific Software Development GmbH, Berlin, Germany, https://atlasti.com). Constant comparative analysis was used to identify themes from the data by inductive category coding and simultaneous comparison of all meaningful units [21]. Codes were generated by the first author (KC) and validated by the second author (SW). Themes and sub-themes were developed and refined by discussion between KC and SW. The full research team reviewed and agreed the final themes, which were subsequently validated by two randomly selected participants from each focus group.

## Results

Three focus groups were undertaken, 2 in Australia and 1 in New Zealand. Seventeen health professionals were recruited in total, the majority being Caucasian women working in the public sector (*n* = 13, 77%) (Table 2). The mean (SD) number of years of clinical experience of managing people with PsA among the rheumatologists was 13.9 (7.9) years and for the allied health professions was 13.2 (6.1) years, (range from 3 to 30 years). Each focus group lasted approximately 60 min.

**Table 2:** Demographic characteristics (*n* = 17). Data presented as number (%) unless specified

Variables	Value
Female	9 (53%)
Ethnicity	
Caucasian	12 (71%)
Chinese	4 (24%)
Indian	1 (6%)
Occupation	
Rheumatologist	10 (59%)
Rheumatologist registrar	2 (12%)
Podiatrist	3 (18%)
Physiotherapist	1 (6%)
Rheumatology care coordinator	1 (6%)
Clinical experience, years, mean (SD)	12.4 (7.5)
Geographical location	
Sydney, Australia	12 (71%)
Auckland, New Zealand	5 (29%)
Health sector	
Public sector	13 (77%)
Private sector	4 (24%)

Three broad themes underpinning suboptimal foot disease management were derived from the data (Table 3). Exemplars were identified from the transcripts to support each theme

**Table 3:** Emergent themes from the focus groups with health professionals

Emergent themes	Sub-themes
1. Missed opportunities and diagnostic delay	• Lack of recognition of foot problems by health professionals • Lack of patient knowledge relating to foot problems • Socioeconomic disparities in care
2. Challenges related to the management of foot problems in PsA	• Varied and fluctuating clinical presentations of PsA • Complexity of foot examination • Lack of appropriate training and knowledge across professions for the management of foot health problems associated with rheumatic disease
3. Lack of specialist podiatry service provision	• Lack of specialised podiatrists working within multidisciplinary rheumatology teams • Lack of allied health professionals with a specialist interest and expertise in inflammatory arthritis • Perceived patient dissatisfaction with limited scope of podiatry practice and high cost of ineffective treatments such as foot orthoses

### Theme 1: Missed opportunities and diagnostic delay

Diagnostic delays in those presenting at disease onset with foot problems were reportedly due to patients either not seeking early medical attention or that foot problems were initially mistaken by health professionals as non-inflammatory musculoskeletal conditions. The health professionals recognised that a PsA diagnosis depends, in part, on whether a GP, allied health professional or dermatologist has the specific knowledge and skill to recognize the symptoms and promptly refer the patient to a rheumatologist. There was a general consensus amongst the rheumatologists that more information about PsA should be provided to people with psoriasis attending dermatology clinics.

*“…they’ll be sent through by musculoskeletal physicians who have been treating for tendonitis thinking that’s due to injury” (rheumatologist 9).*

An earlier diagnosis was described in relation to achieving better disease outcomes and was associated with patients attending private rheumatology practice, having a higher socioeconomic status and presenting with acute inflammatory foot involvement.

*“…the patients I see in (private practice) would be presenting much earlier because they’re more likely to go and speak with their GP sooner about problems and they have the resources to get into see a specialist quickly. So they come when they’re in their early inflammatory phase” (rheumatologist 6).*

The rheumatologists identified the focus of consultations to be on the medical management of PsA. Although it was recognised that some patients may not mention foot problems during rheumatology consultations, nearly all the rheumatologists agreed that they would not routinely ask about or assess the feet unless the patient reported having foot and ankle symptoms. This combined with the perceived view that many patients fail to disclose foot problems to the rheumatologists suggests that the opportunities for diagnosis of foot involvement and referral to podiatry services are being missed.

*“…it’s what’s most important because they might feel like they’ve got 5 minutes to tell you. So they’ll come in with a list of things they want to tell you…So it depends…if everything else is going really well but this is the biggest thing at the moment they might mention it” (rheumatologist 5).*

### Theme 2: Challenges related to the management of foot problems in PsA

Foot pain in PsA was described in relation to global disease activity, local disease activity and/or mechanical pathology, and given this potential for diverse clinical presentations it was acknowledged to be challenging to assess and manage. Frequent reference was made to active foot disease persisting in many patients, despite achieving tight control of their disease with pharmacological treatment.

*“The big difference with psoriatic arthritis is its periodicity and unpredictability so that people at times can do things and at other times it’s really difficult” (rheumatologist 13).*

*“But once it’s established [foot involvement] it’s just hard…It’s probably one of the hardest things to treat” (physiotherapist 3).*

*“…it’s such a heterogeneous disease…it’s a strange beast this disease” (rheumatologist 14).*

The most commonly highlighted barriers to the assessment of the foot in PsA by rheumatologists during consultations included high disease burden leading to time constraints; low priority of foot disease attributed by the patient; the complexity of foot assessments compounded by lack of training; and a lack of opportunity for onward referral. Difficulty with clinical examination of feet and ankles amongst the rheumatologists was attributed to the complex nature of the structure and function of foot anatomy and the interpretation of swelling in the presence of gravitational oedema and obesity. Further training on clinical and image-based foot examinations was deemed important in order to improve identification of pathologies that would benefit from appropriate referral and intervention.

*“The feet I think are architecturally a bit complex and not always as straight forward as hands” (rheumatology registrar 11).*

*“You know us rheumatologists…we are not really trained at all in terms of the functional, like the ankle or the heel and various things… like how the foot should work or take off” (rheumatologist 5).*

Health professionals reported that patients had difficulty with describing and localising foot pain, which appeared to be related to the fluctuating nature of symptoms and being unable to distinguish between joints and skin related symptoms. This further contributed to the difficulty of identifying and assessing foot problems.

*“If you ask them to point they use their whole hand and sort of go ‘Oh here’ over like 80% of the surface of the foot. Even that’s hard so you’ve often got to focally press and find where they wince or are tender and that can help narrow down” (rheumatologist 16).*

An additional barrier to the identification of local inflammatory features in the foot, reported by rheumatologists in New Zealand, was having limited access to imaging and to the expertise required to accurately interpret the findings.

*“Here most of us don’t use that… we don’t use it routinely [Musculoskeletal diagnostic ultrasound]. We had a machine but it’s been taken away” (rheumatologist 9 – New Zealand).*

*“…quite often the report will come back on ultrasound inter-metatarsal bursitis, what does that mean? A bit of fluid here and there you know so it’s quite non-specific” (rheumatologist 9 – New Zealand).*

*“We don’t actually really know what the normal range is that’s the problem” [Musculoskeletal diagnostic ultrasound] (rheumatologist 12 – New Zealand).*

*“I just haven’t got my head around what the normal is for MRI is of the feet” (rheumatologist 13 – New Zealand).*

### Theme 3. Lack of specialist podiatry service provision

One of the major barriers to rheumatologists performing foot examinations was the lack of access to specialised podiatry services in both public hospitals and private clinics.

*“I’ve heard before people will say ‘Why ask patients about their feet because I’ll uncover something that I actually can’t help with’. So why open Pandora’s box effectively” (podiatrist 7).*

*“…sometimes I wonder if we sort of give up a bit. In our hearts because yeah what’s the point of assessing when we can’t do anything about it…we’re not the experts on feet, we are you know we’re rheumatologists, but we don’t necessarily have access to the experts” (rheumatologist 6).*

It was identified by health professionals in Australia and New Zealand that whilst adequate podiatry service provision had been made in the public health system for people with diabetes who have foot problems, only a few high risk foot clinics would accept people with inflammatory arthritis-related foot problems.

*“I do find this quite bizarre that you’re really much better off to have diabetes if you’re going to have bad feet…I’ve had to watch people being shown the door with big ulcerations because they don’t have diabetes, which does seem a bit potty doesn’t it” (podiatrist 8).*

The lack of a multi-disciplinary team approach to preventative care, effective intervention and patient-centred management of PsA was a key topic of discussion during the focus groups, which revealed feelings of frustration. Lack of podiatrists and physiotherapists (in both public and private sectors) with specialist interest, training and knowledge in inflammatory arthritis was a problem reported by the rheumatologists signposting to professional foot care.

*“I find it hard to find the right podiatrist… I don’t know that any of them really specialise in inflammatory foot conditions. But finding someone with an interest in inflammatory arthritis is very difficult” (rheumatologist 6).*

Other barriers preventing uptake of podiatry services by people with PsA perceived by the health professionals were financial constraints and dissatisfaction with podiatry care received based on ineffective treatment and/or unfulfilled expectations due to limited scope of practice. Whilst there was awareness amongst the podiatrists of limited extended-scope practice, ineffective foot care was also linked to the limited evidence to date for non-pharmacological interventions for foot disease in PsA.

*“Yes, a lot of podiatrists would just simply scrape some hard skin down and perhaps don’t have enough insight to be able to comprehensively assess patients as well” (podiatrist 7).*

## Discussion

This is the first study to explore the views of health professionals involved in the management of foot problems in people with PsA. The results indicate that foot health needs were not being fully met due to deficiencies in the diagnosis, assessment and treatment of foot problems related to PsA in Australia and New Zealand. A recent expert review stated that the identification and treatment of PsA were still not optimal [1], which suggests that unmet needs in the management of PsA is a much broader problem. The finding of suboptimal foot disease management in the current study may help to explain the reported persistence of active inflammation in the foot and ankle with a lack of specialist foot care for early detection and tight control of the disease.

Focus group discussions identified that detecting early signs of PsA in the foot was challenging for health professionals due to a lack of awareness about the disease. This study finding supports previous reports of significant delays in PsA diagnosis [5, 7]. Contributing factors to the under-diagnosis of PsA reported in previous studies are the failure to connect skin and joint symptoms and the difficulty in differentiating between inflammatory arthritis and mechanical joint pain [1, 4].

Foot examination during routine rheumatology consultations was reported to be variable in this study, despite the recognition among health professionals of disease persistence in the feet. This finding is consistent with our knowledge of foot problems being overlooked in other rheumatic conditions [18, 22]. Early identification of foot involvement in PsA is of clinical importance as this has been shown to be a predictor for joint damage [23]. Evidence-driven recommendations state that the full 66–68 joint count be used routinely to assess people with PsA, as significant proportions of active disease can be missed in the feet and hands [1, 24]. No guidelines exist for foot assessment in PsA and the omission of many anatomical sites in the foot and ankle from standard clinical indices, may lead to active disease in the foot being missed along with the opportunity to prevent joint damage.

The complexity of foot examination has been acknowledged within the PsA literature and is partly due to the heterogeneity of clinical symptoms [9, 10, 23]. This study identified the need for specialist training of podiatrists and rheumatologists to develop advanced skills for managing foot health in rheumatic disease. Inadequate podiatry service provision in the public health system reported in this study may in part explain the lack of foot care specialists in the rheumatology field because health service demand typically drives training need. Postgraduate training courses have been implemented in the UK in response to such concerns within the wider rheumatology community [12]. This model may need to be expanded in Australia and New Zealand to facilitate knowledge transfer between rheumatologists and allied health professionals in the absence of multidisciplinary rheumatology teams.

Establishing locally representative data on the challenges of foot disease management in PsA is an important step towards improving management approaches in the future. Limited information from European studies on the provision of health care for foot disease in PsA [8] may not translate to other countries, due to differences in health care structure, organisation and provision. Although previous research has focused on local RA foot care provision [13, 15], PsA is a distinct disease entity with different challenges associated with disease management, which is supported by the current study themes.

Difficulty experienced by patients with describing and localising symptoms in the foot and ankle was recognised by the health professionals in this study. Although this issue has been previously highlighted in RA [25], it has heightened relevance in PsA with previous studies demonstrating that patients have difficulty distinguishing between the musculoskeletal and dermatological components of their disease [26]. To facilitate foot pain self-report and localisation, foot manikins have been utilised in both clinical practice and population-based research [27–29]. However, it is not known how accurately pain locations are transferred on to foot manikins by people with PsA-related foot pain. Routine use of musculoskeletal diagnostic ultrasound in clinical practice would further optimise the identification of localised disease activity in the foot in PsA, and the health professionals in this study highlighted the training and development of expertise required to accurately interpret image-based findings.

Key concepts regarding foot disease management in PsA comprise reducing diagnostic delay, improving knowledge and awareness among patients and health professionals and increasing specialist podiatry service provision. A better understanding of disease persistence in the foot in PsA is required to inform the direction of future research in this area. Future work involves implementation of a survey to obtain information about foot involvement from people with PsA, generating population-based data for Australia and New Zealand. Early identification of foot and ankle problems using a screening tool or outcome measure specific to PsA may help to prevent non-disclosure of foot problems by patients and promote more timely referral and intervention. Currently there are no validated outcome measures specific to foot involvement and its impact in PsA, which limits our understanding of foot disease in PsA and impedes definitive strategies for ‘tight control’ of disease activity in the foot. Further work to validate the use of foot manikins in PsA may help to improve foot pain reporting by patients.

This study was preliminary and exploratory in nature, involving a small number of participants. As such, the findings may not be representative of health professional in other regions of Australia and New Zealand. However, the small sample size provided the opportunity for deeper exploration within a qualitative paradigm. Rheumatology nurses and dermatologists were invited to take part in the study but were unable to attend, resulting in an under representation of these professional groups. The views of people with PsA-related foot problem were not sought in this current study as the focus was to explore experiences related to foot health assessment and management from the perspective of rheumatology health professionals working with this patient group. Future work on patient’s views is required.

## Conclusions

This study has generated preliminary evidence that suggests the identification and management of PsA-related foot involvement may potentially be suboptimal in Australia and New Zealand. Further work is required to investigate the nature and extent of foot involvement and related impacts from the patient perspective, and to further examine current foot care deficiencies in PsA with a view to remediation.

## Abbreviations

PsA: Psoriatic arthritis
RA: Rheumatoid arthritis
